# Interdependence of dental parameters, mandibular mobility and static postural sway as well as load distribution in male subjects of advanced working age

**DOI:** 10.1007/s00784-026-07193-z

**Published:** 2026-10-03

**Authors:** Christian W., Krüger D., Erbe C., Maurer-Grubinger C., Oremek G., Groneberg D.A., Ohlendorf D.

**Affiliations:** 1 Institute of Occupational, Social and Environmental Medicine, Goethe University Frankfurt, 60596 Frankfurt am Main, Germany; 2 Department of Orthodontics and Dentofacial Orthopedics, University Medical Center of the Johannes Gutenberg-University, Mainz, Germany

**Keywords:** Dental occlusion, Temporomandibular system, Postural control, Middle-aged men, Axiography

## Abstract

**Background:**

Maintaining static postural sway as well as load distribution is an essential component of overall physical stability in older age. The temporomandibular system is a measurable influencing factor due to functional interdependencies, although it has not yet been sufficiently investigated with regard to sex- and age-specific differences. This cross-sectional analysis therefore aims to examine the association between occlusion, mandibular mobility and static postural sway as well as load distribution in men aged 51 to 60, taking into account body mass index (BMI) and mandibular mobility.

**Methods:**

A total of 98 healthy men, aged between 51 and 60 years (mean age: 55.9 ± 2.72 years), volunteered for the study. Their mean BMI was 27.01 ± 3.91 kg/m² (mean height: 1.81 ± 0.07 m; mean weight: 88 ± 14.13 kg). BMI classification was based on WHO criteria. Postural measurements were conducted using a pressure platform. Mandibular movements (laterotrusion, protrusion and mouth opening) were recorded using an axiograph. Additionally, a model analysis according to Schopf was performed (midline deviation, supporting zone, and transverse width differences in the maxilla and mandible on both the right and left; occlusion of the canines [3s] and first molars [6s] on both sides; overjet; and overbite). The significance level was set at 5%.

**Results:**

Dental model analysis revealed that no participant was classified as Angle Class I, (mean overjet 3.93 mm and mean overbite 3.44 mm). All subjects exceeded the normal ranges defined by the Schopf classification, and the majority (75%) presented with Angle Class II.

No significant association was found between BMI and static postural sway and well as load distribution in the overall analysis (p > 0.05).

BMI subgroup analysis: In the normal-weight group, occlusion of the right first molar (6) exhibited a modest positive correlation with ellipse area (p ≤ 0.01), while occlusion of the right canine (3) exhibited a modest positive correlation with frontal sway (p ≤ 0.01). In the pre-obese group, only a weak negative correlation was observed between occlusion of the right first molar (6) and forefoot load (p ≤ 0.01). In the obese group, occlusion of the right canine (3) showed a strong positive correlation with rearfoot load (p ≤ 0.01) and right laterotrusion showed a significant correlation with sagittal sway (p ≤ 0.02; moderate). Mandibular mobility subgroups in relation to BMI: in participants with hypomobile mouth opening, BMI correlated positively with frontal sway (p ≤ 0.03, strong). In the group with normal right laterotrusion, increasing BMI was associated with significant increases in frontal and sagittal sway, as well as ellipse parameters (p ≤ 0.01–0.05; all weak). In individuals with hypermobile mandibular function, BMI showed a strong negative correlation with occlusion of the first molars (6) in the left forefoot (p ≤ 0.001), as well as a strong positive correlation with the left rearfoot (p ≤ 0.01).

**Discussion:**

While mandibular mobility parameters (laterotrusion, protrusion, mouth opening) were predominantly within normal limits, dental model analysis revealed that the majority (75%) presented with Angle Class II. Additional exploratory subgroup indicated that deviations in canine and molar occlusion were associated with greater body sway and larger sway ellipses. An increased BMI does not appear to directly cause instability but may be associated with, or exacerbate, pre-existing functional limitations or occlusal dysfunctions. Therefore, clinical assessment should adopt a holistic approach that integrates BMI with individual constitutional, occlusal, and functional parameters.

**Supplementary Information:**

The online version contains supplementary material available at https://doi.org/10.1007/s00784-026-07193-z.

## Background

Maintaining physical mobility and postural control is particularly important for older men, many of whom remain in full-time employment in Germany beyond the statutory retirement age of 67 [1–7, 29]. The maintenance of postural stability is a critical factor in physical resilience, particularly as age-related changes affect sensorimotor regulation, thereby counteracting age-related changes and disease progression, is well established [8–10]. These factors help individuals to meet continuously increasing qualitative and quantitative demands, including those in the workplace [11–14]. In this context, another potentially relevant factor, especially with regard to resilience to occupational stress [15], is the temporomandibular system. Due to its neurofunctional capacities, it can act as a form of ‘valve’ for reducing or relieving stress, as psychological and emotional tension can be transferred to the teeth and surrounding tissues through bruxism [16]. Minor disturbances are often compensated for by the body, particularly via the cervical and neck musculature, as well as through local sensorimotor regulation involving the temporomandibular joint [11, 17, 18]. Due to the functional interdependence between motor control of posture and movement, and mandibular position, these loads can have a significant impact on postural control [16, 19–21].

Several systematic reviews have investigated these relationships. Cezar Ioniță et al. [22] generally confirmed that various occlusal positions influence postural control, the most frequently assessed parameter compared to other components. Similarly, Solano et al. [23] reported that mandibular positions predominantly induced postural changes in the mediolateral direction, whereas malocclusions primarily affected the anteroposterior axis. Furthermore, Stancker et al. [24] concluded that malocclusion (an imbalance between the masticatory and neuromuscular systems) can have a negative impact on body posture and postural parameters. Malocclusion can also influence the function of the temporomandibular system, which transmits information to the vestibular system that is involved in regulating postural balance. Disruption to one of the main sensory systems (such as the feet, eyes or temporomandibular system) may therefore result in altered posture.

Nowak et al. [25, 26] and Alessandro et al. [27] observed a significant tendency towards anterior displacement of the centre of pressure (CoP) in young adults with Angle Class II, along with a significantly more anterior head position and increased cervical and lumbar lordosis, compared to individuals with Angle Classes I and III. In contrast, participants with Angle Classes I and III showed posterior displacement of the centre of pressure (*p* < 0.001).

The aforementioned systematic reviews [22–25], which examined the associations between occlusion and postural control, either lacked sex-specific analyses or considered them to be of minor importance. Furthermore, they did not take into account age-related neurophysiological changes or the potential influence of body mass index (BMI). However, sex-specific differences become apparent from around 12–13 years of age and can be attributed to neurophysiological and hormonal differences in development [28–32]. Age-related neurophysiological changes also affect coordination abilities.

The relationship between permanent dental parameters, mandibular mobility, and postural control in relation to age and gender has already been investigated in women aged 21–30 and 41–50 [33–35]. In the younger group, a higher BMI was associated with increased forefoot loading, albeit with a small effect size. Participants with greater forefoot loading also exhibited increased mouth opening, but no changes in protrusion or laterotrusion [33, 34]. In women aged 41–50, restricted right laterotrusion was associated with reduced forefoot loading and increased rearfoot loading. Meanwhile, excessive left laterotrusion was associated with reduced frontal sway, as well as a smaller sway ellipse in terms of area, height, and width [32].

BMI is frequently analysed as a negative influencing factor on postural control in addition to age and sex [36–38]. Individuals with a higher BMI typically have greater body mass in the thoracic and abdominal regions, which leads to an anterior shift in the centre of pressure [38, 39]. Increased load may therefore impair balance stability [36], with men additionally exhibiting specific instability in the mediolateral axis [40, 41]. Conversely, Teasdale et al. demonstrated that weight loss linearly improves postural control. The observed sex differences may be partly explained by the prevalence of abdominal obesity in men [36, 37]. Overall, these findings suggest that a higher BMI negatively affects postural control. It should also be noted that obesity in adults can lead to reduced mobility and functional limitations [39, 42], which in turn increase the risk of falls — an especially important issue as we age.

The aim of the present analysis, conducted as part of a larger project [43], is to investigate the relationship between occlusion, mandibular mobility and static postural sway as well as load distribution in men aged 51 to 60, while taking BMI [34, 35, 44] and mandibular mobility [45, 46] into account. This age group is of particular interest because labour force participation in Germany exhibits a clear gender-specific pattern: the traditional male breadwinner model still dominates the German labour market [4, 47–49], meaning men are more likely to be employed full-time than women [1–4, 29]. Furthermore, in high-income countries, including Germany, men often continue working beyond the current statutory retirement age of 67 [5–7], and an increase to 68 has been under political discussion for some time [50–54].

## Methods

### Participants

A total of 98 healthy men aged between 51 and 60 years (mean: 55.9 years; median: 56 years) voluntarily took part in this study. All participants were recruited from two geographically distinct general dental practices.

The proportion of right-handed individuals was 86%. On average, participants were 1.81 ± 0.07 m tall and weighed 88 ± 14.13 kg, resulting in a mean BMI of 27.01 ± 3.91 kg/m² according to the WHO classification [55, 56]. According to WHO criteria [55], 36 men were of normal weight (group II; 18.50–24.90 kg/m²), 45 were overweight (group III; 25.00–29.90 kg/m²), and 17 were obese (group IV; ≥30.00 kg/m²). In total, 84 participants were right-handed, 10 were left-handed and four were ambidextrous.

Twenty-one (21.42%) of the 98 participants had completed orthodontic treatment. The distribution of Angle classes was as follows: *n* = 0 for Class I, *n* = 75 for Class II and *n* = 23 for Class III.

Regarding physical activity, 44 participants reported engaging in regular sports activities, while 54 reported little to no regular exercise. Occupational activity varied: Forty-six men (46.94%) reported predominantly sedentary work (e.g. office employees); 44 men (44.89%) reported predominantly standing occupations (e.g. hairdressers); and eight men (8.17%) reported physically active jobs involving substantial movement (e.g. carpenters or industrial mechanics).

At baseline, all participants completed a medical history questionnaire from the Centre for Dentistry, Oral and Maxillofacial Medicine at Goethe University Frankfurt am Main (Germany). This questionnaire covered general conditions such as diabetes mellitus, tinnitus, osteoporosis and rheumatic diseases. It also included questions on acute joint and musculoskeletal pain, headaches and migraines, acute temporomandibular joint (TMJ) pain or sounds, accidents or surgeries affecting the musculoskeletal system or TMJ in the past two years, a deep bite of more than 3 mm and current medication use.

Only participants who subjectively considered themselves to be healthy and did not report any of the predefined criteria in the questionnaire were included in the study. The classification of participants as healthy was based on self-reported information. Specifically, participants reported no acute symptoms and subjectively considered themselves to be in good health at the time of assessment.

Participants who reported occasional TMJ sounds or pain underwent a brief clinical examination (palpation) to assess the likelihood of TMJ disorders, which constituted a strict exclusion criterion. Further exclusion criteria address other relevant dental factors, including fixed prosthodontic restorations (e.g., crowns, bridges, and implants), missing teeth, and extensive restorative treatments. Moderate musculoskeletal complaints were permitted provided they did not interfere with daily activities. No radiation-based imaging (e.g. X-rays or CT scans) was performed to verify spinal health or subjective reports, as unnecessary radiation exposure is considered unethical in Germany (ALARA principle) [57–59]. Combining the use of this medical history questionnaire with additional questions on completed orthodontic treatment and physical activity [60] resulted in a study population that was considered to be free of acute health symptoms at the time of data collection.

The rights of the participants were protected, and all subjects were thoroughly informed about the study design before providing written informed consent. The study was approved by the local ethics committee of the Medical Faculty of Goethe University (No. 103/16) and was conducted in accordance with the 1964 Declaration of Helsinki and subsequent amendments.

### Measurement systems

#### Posturography

A pressure measurement platform (GP MultiSens, GeBioM, Münster, Germany) was used to record load distribution and body sway. Measurements were taken over a surface area of 38.5 × 38.5 cm containing 2,304 resistive sensors (two sensors per cm²), each measuring 8 × 8 mm. At a sampling rate of 200 Hz, the pressure generated by loading was recorded in a matrix arrangement of sensor units. Pressure changes were detected via high-impedance amplifiers (unit: pascal, force per unit area), with signals captured through 48 connections arranged in rows and columns. According to the manufacturer’s specifications, the potential measurement error is ± 5%.

During data acquisition, all participants stood barefoot on the platform in their habitual posture with their eyes open and their jaw relaxed. Three trials of 30 s each were recorded per participant. The mean value across the three trials was calculated for each postural parameter and used in all subsequent analyses.

#### Axiography

Three-dimensional mandibular movements were recorded using the ARCUS DIGMA SD II system (KaVo/Zebris Medical GmbH, Biberach, Germany), which uses ultrasound. This system comprises a 250 g facebow (340 g including sensors) and a 100 g transmitter, which is attached to a mandibular bite fork (paraocclusal tray) via magnets. The bite fork is fixed to each participant individually using bite registration material (Luxabite, DMG).

A 40 kHz ultrasound navigator, aligned in three dimensions, recorded spatial positional changes at a sampling rate of 50 Hz. According to the manufacturer, the measurement accuracy ranges between ± 0.1 and 0.2 mm, ensuring clinically relevant reproducibility [60–63]. The recorded data were processed using the associated WinJAW+ software (version 1.0).

#### Model analysis

For this, impressions of the maxilla and mandible were taken using alginate (Trealgin Chromatic, Schütz Dental, Rosbach vor der Höhe, Germany). These impressions were subsequently cast in dental stone (Natura, DIN EN ISO 6873, Type 3, Siladent, Dr. Böhm & Schöps GmbH, Goslar, Germany).

To register the individual habitual occlusion, modelling wax (Standard Modelling Wax 175/80, 1.25 mm, Gebrüder Steinhart Wachswarenfabrik GmbH & Co. KG) was used to relate the model segments.

A model analysis was performed for quantitative assessment of occlusion (alginate impressions, plaster models and measurements using a ‘Beerendonk’ caliper). This precision instrument, which is commonly used in orthodontics and dental technology, provides measurement accuracy to 0.1 mm [64]. Classification of occlusal parameters followed the criteria described by Schopf [65, 66]. To ensure measurement reliability, the examiner completed a structured calibration protocol prior to data collection, following established standards for plaster model analysis [67, 68]. Intra-examiner reliability was assessed by a single examiner who repeated the measurements on 10 randomly selected dental models after a 14-day interval. The intraclass correlation coefficient (ICC) was 0.93.

### Evaluation parameters

Postural control: percentage distribution of load (%) across all four quadrants (left/right forefoot and rearfoot), as well as aggregated values for the left/right foot and forefoot/rearfoot. Additionally, maximum frontal and sagittal displacement of the center of pressure (mm), and the width (cm), height (cm), and area (cm²) of the sway ellipse were analyzed.

The relevant evaluation parameters of axiography and model analysis, including their classifications according to Schopf [65, 66], are listed below.

**Table:** 

Axiography		
Laterotrusion left/right (mm)	Normal: 7–12 mm Hypofunctional: < 7 mm Hyperfunctional: > 12 mm	
Protrusion (mm)	Normal: 7–11 mm Hypofunctional: < 7 mm Hyperfunctional: > 11 mm	
Mouth Opening (mm)	Normal: 40–60 mm Hypofunctional: < 40 mm Hyperfunctional: > 60 mm	
Model Analysis		
Midline Deviation (Upper/Lower Jaw, mm)	1. No deviation: Reference value = 0 mm (symmetrical baseline) 2. Deviation to the left: < 0 mm, negative values (hypofunctional) 3. Deviation to the right: > 0 mm, positive values (hyperfunctional)	
Transverse Width Difference (Upper/Lower Jaw, mm)	1. Normal: Symmetrical dental arch (actual width ≈ target width) 2. Crowding/Narrow Arch: Actual width < target width 3. Spacing/Wide Arch: Actual width > target width	
Occlusion of Canines (3s)/First Molars (6s) (bilateral, mm)	1. Class I – multipoint contact occlusion (neutral) 2. Class II – distal occlusion 3. Class III – mesial occlusion	
Occlusal Assessment		
Hyperocclusion	Excessive contact, potential local interference point. (Right first molar > 2 mm)	
Normal Occlusion	Within the physiological tolerance range. (− 2 mm ≤ right first molar ≤ 2 mm)	
Hypoocclusion	Reduced contact or gap formation. (Right first molar < − 2 mm)	
Dental Measurements		
Overjet (mm)	Horizontal distance between the labial surfaces of the upper and lower anterior teeth. Normal range: 2.1–2.9 mm Increased: > 3 mm	
Overbite (mm)	Vertical overlap of the lower anterior teeth by the upper anterior teeth. Normal range: 1–3 mm Deep bite (increased overbite): > 3 mm	

### Statistical analysis

An a priori sample size estimation was performed based on the primary objective of investigating associations between dental parameters, mandibular mobility, static postural sway, and plantar load distribution in healthy men aged 51–60 years. A target sample size of approximately *n* = 100 participants was defined. For correlation analyses, a sample size of approximately *n* = 100 provides 80% power to detect correlation coefficients of *r* ≥ 0.25 (α = 0.05), corresponding to at least a weak association according to Evans’ classification.

The final sample consisted of *n* = 98 participants, as five initially available datasets had to be excluded due to incomplete data for relevant parameters required for the analyses. The achieved sample size was therefore considered adequate for the primary correlation analyses. However, subsequent stratified subgroup analyses (e.g., according to BMI categories) resulted in smaller subgroup sizes. These analyses were considered exploratory and were interpreted with caution due to the limited statistical power of individual subgroups.

Statistical analyses were performed using the software package BIAS (version 10). Initially, data were tested for normal distribution using the Kolmogorov–Smirnov goodness-of-fit test. Descriptive statistics were calculated for all variables, including the median, first and third quartiles (Q1–Q3), tolerance ranges, and 95% confidence intervals (CIs). As the data were predominantly not normally distributed, non-parametric statistical methods were applied. Differences between groups were assessed using the Kruskal–Wallis test, Wilcoxon matched-pairs test, Wilcoxon–Mann–Whitney U test, and Conover–Iman post hoc test, as appropriate. In cases where subgroup sizes were small (*n* < 10), these groups were excluded from inferential statistical analyses due to limited statistical reliability p-values obtained from multiple group comparisons were adjusted using the Bonferroni–Holm correction.

Correlations were calculated using Spearman’s rho and Kendall’s tau. Effect sizes (rho) were interpreted according to Evans as follows: <0.2 (poor), 0.2–0.4 (weak), 0.4–0.6 (moderate), 0.6–0.8 (strong), and > 0.8 (optimal). Due to the exploratory nature of the correlation analyses, no adjustment for multiple comparisons using the Bonferroni–Holm correction was applied.

The level of significance was set at 5%.

This study was reported in accordance with the Strengthening the Reporting of Observational Studies in Epidemiology (STROBE) guidelines for cross-sectional studies.

## Results

### Anamnestic parameters

Table 1 shows the mean, standard deviation, median, first and third quartiles, tolerance range (lower and upper limits) and confidence intervals (lower and upper bounds) for the anamnestic parameters (age, height and weight) and postural control parameters.

**Table 1:** Mean, standard deviation, median, first and third quartiles, tolerance range (lower and upper limits), and confidence interval (lower and upper bounds) of the anamnestic parameters (age, height, weight) and postural control parameters

	Mean	Standard deviation	Median	1. Quartil	3. Quartil	Tolerance range lower upper bounds bounds		Confidence interval left right bounds bounds	
Height (m)	1.81	0.07	1.80	1.77	1.87	1.65	1.96	1.79	1.82
Weight (kg)	88.00	14.13	87.00	78.0	96.00	59.91	117.51	85.81	91.60
BMI (kg·m⁻²)	27.01	3.91	26.17	24.33	29.33	21.34	36.97	26.23	27.79
Forefoot Left (%)	20.68	7.37	20.0	15.58	24.0	8.47	39.01	19.20	22.15
Forefoot Right (%)	17.11	6.95	16.83	12.16	20.58	3.24	30.97	15.71	18.50
Hindfoot Left (%)	30.09	7.51	31.0	26.58	34.41	10.82	44.19	28.58	31.60
Hindfoot Right (%)	32.29	8.47	32.0	26.58	39.08	15.40	49.49	30.60	33.99
Left (%)	50.85	8.60	51.0	46.66	55.49	31.67	69.20	49.12	52.57
Right (%)	49.27	8.61	49.16	44.50	53.58	30.79	68.33	47.54	50.99
Forefoot (%)	37.84	10.50	36.16	29.91	44.83	21.49	60.55	35.73	39.95
Hindfoot (%)	62.00	10.27	63.67	55.24	69.49	39.44	78.5	59.94	64.06
Frontal sway (mm)	14.18	6.60	12.33	9.50	19.33	5.15	31.54	12.84	15.52
Sagittal sway (mm)	20.13	8.80	18.16	14.00	23.00	7.63	44.38	18.36	21.89
Ellipse area (cm²)	2.24	1.86	1.63	0.95	2.66	0.32	7.73	1.87	2.62
Ellipse width (cm)	1.16	0.49	1.08	0.80	1.44	0.44	2.36	1.06	1.26
Ellipse height (cm)	0.52	0.23	0.46	0.38	0.65	0.18	1.08	0.47	0.57

No significant correlations were found between postural control parameters and BMI (*p* ≥ 0.05). When comparing the three BMI categories (normal weight, pre-obese and obese) across all postural control parameters, no significant group differences were observed.

No significant correlations were found between postural control parameters and body weight (*p* > 0.05).

Height showed a significant positive correlation with rearfoot load distribution (*p* ≤ 0.04), sagittal sway (*p* ≤ 0.01) and ellipse width (*p* ≤ 0.02), with weak effect sizes in all cases.

Comparisons of physical activity revealed no significant differences between active and inactive participants (*p* > 0.05).

Furthermore, no significant differences in postural control parameters were observed when comparing orthodontically treated men (*n* = 21) and untreated men (*n* = 77), or symmetrical (*n* = 57) and asymmetrical (*n* = 41) Angle classes (*p* > 0.05).

### Mandibular movements

Table 2 shows the mean, standard deviation, median, first and third quartiles, tolerance range (lower and upper limits) and confidence intervals (lower and upper bounds) for right and left laterotrusion, protrusion and mouth opening. The median for right laterotrusion was 9.1 mm (range 6.4–10.92 mm), and for left laterotrusion it was 10.05 mm (range 7.47–11.32 mm).

**Table 2:** Mean, standard deviation, median, first and third quartiles, tolerance range (lower and upper limits), and confidence interval (lower and upper bounds) of the axiographic parameters

	Mean	Standard deviation	Median	1.Quartil	3.Quartil	Tolerance range lower upper bounds bounds		Confidence interval Left Right bounds bounds	
Laterotrusion right (mm)	8.80	3.19	9.10	6.40	10.92	2.43	15.17	8.16	9.44
Laterotrusion left (mm)	9.51	2.85	10.05	7.47	11.32	3.09	14.7	8.93	10.08
Protrusion (mm)	9.71	2.33	9.85	7.90	11.12	4.88	14.25	9.24	10.18
Mouth opening (mm)	48.81	6.66	49.50	44.20	54.10	35.52	62.10	47.47	50.15

The length of the protrusion path was 9.85 mm (range 7.9–11.12 mm). The median mouth opening was 49.5 (44.2/54.1) mm.

The correlations between postural and axiographic parameters (mouth opening, protrusion and left and right laterotrusion) were not significant (*p* > 0.05). Group comparisons for mouth opening (normal: 40–60 mm vs. >60 mm), protrusion (normal: 7–11 mm vs. <7 mm vs. >11 mm) and laterotrusion (normal: 7–12 mm vs. <7 mm vs. >12 mm) also showed no significant differences.

**Fig. 1:**
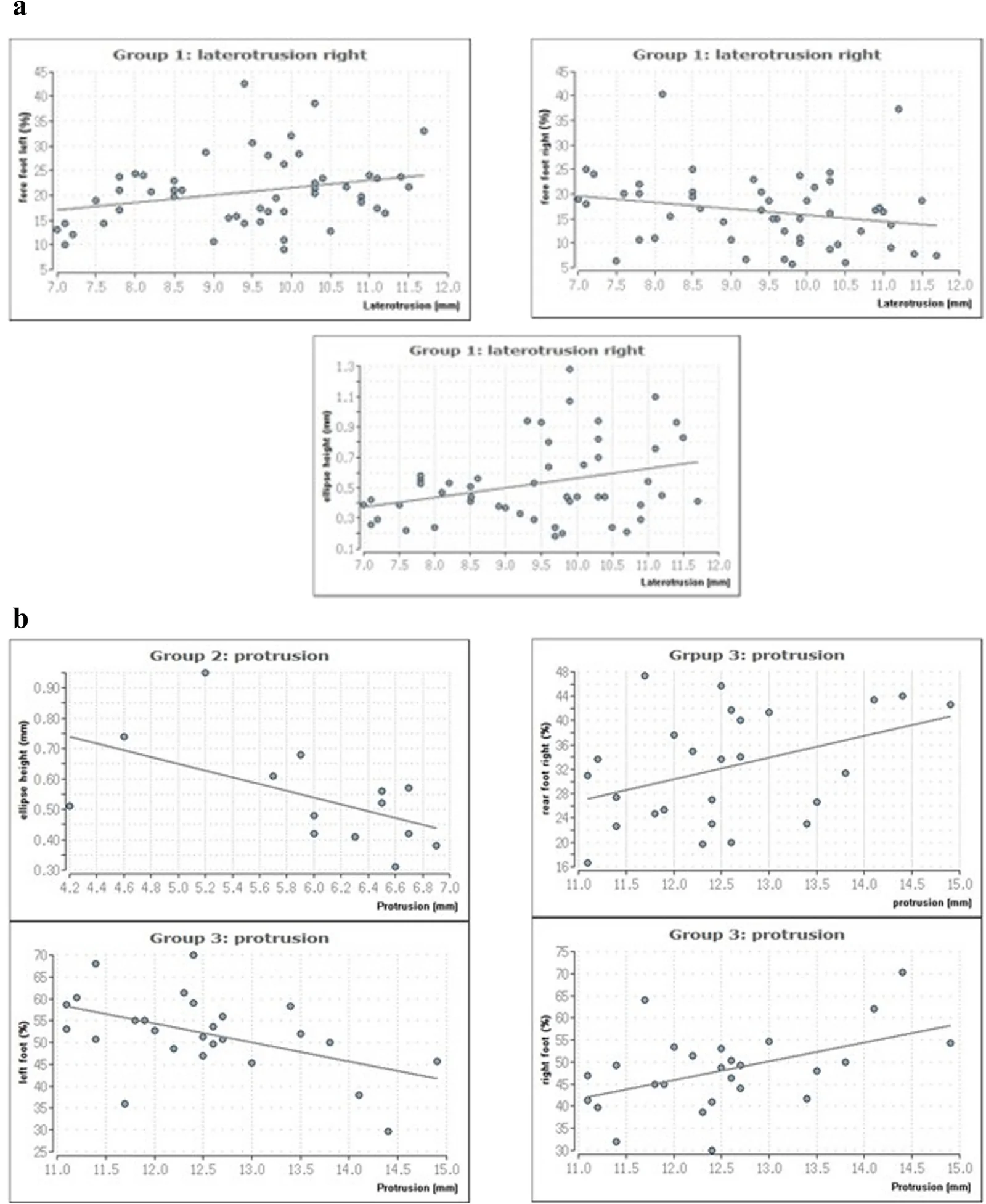
(**a**) Correlation between mandibular movement parameters (axiography) and postural control parameters (posturography) in Group 1 (normal range of laterotrusion). (**b**) Relationship between axiographically measured mandibular movements and postural parameters, differentiated by BMI groups (normal weight, overweight and obesity)

When the categorised mandibular mobility parameters were correlated with the postural control parameters, a significant correlation was found for right laterotrusion in group 1 (*n* = 50), with left and right forefoot load (*p* ≤ 0.04 and 0.05, respectively; rho: 0.29 and − 0.28, respectively) and with ellipse height (*p* ≤ 0.05; rho: 0.27) (see Fig. 1a). No significant correlations were observed in groups 2 and 3 (*n* = 31 and 17, respectively) (*p* > 0.05).

No significant correlations were found for left laterotrusion in any group (G1 = 60, G2 = 20, G3 = 18) (*p* > 0.05).

For protrusion, group 2 (*n* = 14) showed a significant positive correlation between protrusion and ellipse height (*p* ≤ 0.03; rho = 0.59) (see Fig. 1b). Group 3 showed significant correlations with the percentage load of the right and left foot (*p* ≤ 0.01 and 0.02, respectively; rho: −0.48 and 0.44, respectively) and with the right rearfoot (*p* ≤ 0.05; rho: 0.38). No correlations were found in group 1 (*n* = 58) (*p* > 0.05).

No correlations were found for mouth opening in either group (G1 = 87; G2 = 10).

Additional stratification by BMI groups revealed significant, albeit isolated, correlations between postural control and axiographic parameters (Table 3). For mouth opening, a weak significant correlation was found in normal-weight participants (*p* ≤ 0.05; 95% CI 0.01–0.66) (Fig. 1b). Obese participants showed significant correlations with sagittal sway (95% CI −1.00- −0.20), ellipse area (95% CI −1.00- −0.14) and height (95% CI −0.89- 0.09) for right laterotrusion (*p* ≤ 0.01–0.02) with moderate to strong effect sizes (Fig. 1b).

**Table 3:** Correlation between mandibular movement parameters (axiography) and postural parameters, filtered by BMI groups (normal weight, pre-obese, obese)

	Mouth opening						Protrusion					
	Normal weight (*n* = 36)		Pre-obese (*n* = 45)		Obese (*n* = 17)		Normal weight (*n* = 36)		Pre-obese (*n* = 45)		Obese (*n* = 17)	
	p-value	Rho	p-value	Rho	p-value	Rho	p-value	Rho	p-value	Rho	p-value	Rho
Forefoot left	0.28	0.18^1^	0.79	−0.03^1^	0.86	−0.04^1^	0.67	−0.07^1^	0.82	−0.03^1^	0.39	−0.21^2^
Forefoot right	**0.05**	0.33^2^	0.81	−0.03^1^	0.55	0.15^1^	0.45	0.12^1^	0.11	−0.23^2^	0.73	−0.08^1^
Hindfoot left	0.59	−0.09^1^	0.42	0.12^1^	0.38	−0.22^2^	0.28	−0.18^1^	0.27	0.16^1^	0.71	−0.09^1^
Hindfoot right	0.06	−0.32^2^	0.72	−0.05^1^	0.70	0.09^1^	0.26	0.19^1^	0.96	−0.01^1^	0.27	0.28^2^
Left foot (%)	0.92	0.02^1^	0.72	0.05^1^	0.10	−0.39^2^	0.16	−0.02^1^	0.28	0.16^1^	0.47	−0.17^1^
Right foot (%)	0.88	0.02^1^	0.66	−0.06^1^	0.10	0.39^2^	0.15	0.24^2^	0.44	−0.11^1^	0.47	0.17^1^
Forefoot (%)	0.08	0.28^2^	0.20	−0.19^1^	0.58	0.13^1^	0.89	−0.02^1^	0.19	−0.19^1^	0.81	0.06^1^
Hindfoot (%)	0.10	−0.27^2^	0.21	0.18^1^	0.25	−0.28^2^	0.53	0.10^1^	0.19	0.19^1^	0.94	−0.01^1^
Frontal sway	0.64	−0.07^1^	0.82	0.03^1^	0.81	−0.05^1^	0.91	−0.01^1^	0.67	−0.06^1^	0.50	−0.16^1^
Sagittal sway	0.90	0.02^1^	0.90	−0.01^1^	0.63	−0.11^1^	0.66	−0.07^1^	0.37	0.13^1^	0.43	−0.19^1^
Ellipse area	0.98	−0.01^1^	0.85	0.02^1^	0.75	−0.07^1^	0.69	−0.06^1^	0.66	−0.06^1^	0.33	−0.24^2^
Ellipse width	0.71	0.06^1^	0.99	0.001^1^	0.85	−0.04^1^	0.40	−0.14^1^	0.93	−0.01^1^	0.43	−0.20^2^
Ellipse height	0.66	−0.07^1^	0.84	0.03^1^	0.61	0.13^1^	0.87	−0.02^1^	0.57	−0.08^1^	0.86	−0.04^1^

	Laterotrusion right						Laterotrusion left					
	Normal weight (*n* = 36)		Pre-obese (*n* = 45)		Obese (*n* = 17)		Normal weight (*n* = 36)		Pre-obese (*n* = 45)		Obese (*n* = 17)	
	p-value	Rho	p-value	Rho	p-value	Rho	p-value	Rho	p-value	Rho	p-value	Rho
Forefoot Left	0.77	0.04^1^	0.68	−0.06^1^	0.28	−0.27^2^	0.77	0.04^1^	0.53	−0.09^1^	0.23	−0.30^2^
Forefoot Right	0.83	−0.03^1^	0.18	−0.20^2^	0.31	−0.26^2^	0.60	−0.08^1^	0.21	−0.18^1^	0.73	0.08^1^
Hindfoot Left	0.83	−0.03^1^	0.44	0.11^1^	0.28	0.27^2^	0.97	0.005^1^	0.19	0.19^1^	0.74	0.08^1^
Hindfoot Right	0.68	0.07^1^	0.92	−0.01^1^	0.81	0.06^1^	0.72	0.06^1^	0.83	0.03^1^	0.95	−0.01^1^
Left foot (%)	0.93	0.01^1^	0.65	0.06^1^	0.82	−0.05^1^	0.42	0.14^1^	0.77	0.04^1^	0.20	−0.21^2^
Right foot (%)	0.88	−0.02^1^	0.52	−0.09^1^	0.82	0.05^1^	0.39	−0.14^1^	0.92	−0.01^1^	0.39	0.21^2^
Forefoot (%)	0.78	−0.04^1^	0.3	−0.15^1^	0.26	−0.27^2^	0.68	−0.07^1^	0.12	−0.23^2^	0.89	0.03^1^
Hindfoot (%)	0.47	0.12^1^	0.31	0.15^1^	0.31	0.25^2^	0.37	0.15^1^	0.14	0.22^2^	0.98	0.004^1^
Frontal sway	0.61	0.08^1^	0.19	−0.19^1^	0.05	−0.46^3^	0.25	0.19^1^	0.73	−0.05^1^	0.89	0.03^1^
Sagittal sway	0.81	−0.04^1^	0.62	0.07^1^	**0.01**	−0.62^4^	0.68	0.07^1^	0.51	0.1^1^	0.20	−0.03^1^
Ellipse area	0.66	0.07^1^	0.28	−0.16^1^	**0.02**	−0.58^3^	0.38	0.15^1^	0.59	−0.08^1^	0.93	−0.02^1^
Ellipse width	0.81	0.04^1^	0.97	−0.01^1^	**0.02**	−0.40^3^	0.83	0.03^1^	0.87	0.02^1^	0.84	−0.05^1^
Ellipse height	0.74	0.05^1^	0.22	−0.18^1^	0.15	−0.35^2^	0.25	0.19^1^	0.47	−0.10^1^	0.54	0.15^1^

Effect size classification (rho) according to Evans: 1 = < 0.2 (poor); 2 = 0.2–0.4 (weak); 3 = 0.4–0.6 (moderate); 4 = 0.6–0.8 (strong); 5 = > 0.8 (optimal). Significant correlations are marked in bold

Further stratification by mandibular mobility and subsequent correlation between BMI and postural control revealed isolated significant correlations (Table 4).

**Table 4:** Correlation between BMI and postural parameters, filtered by mandibular mobility groups (normal, hypofunctional, hyperfunctional) for each parameter

	Protrusion						Mouth opening					
	Norm (58)		Hypofunctional (14)		Hyperfunctional (26)		Norm (87)		Hypofunctional (10)		Hyperfunctional (1)	
	p-value	Rho	p-value	Rho	p-value	Rho	p-value	Rho	p-value	Rho	p-value	Rho
Forefoot Left	0.78	0.03^1^	0.94	−0.02^1^	0.64	−0.09^1^	0.82	−0.02^1^	0.14	0.53^3^		
Forefoot Right	0.19	0.17^1^	0.63	−0.13^1^	0.70	−0.07^1^	0.62	0.05^1^	0.64	0.18^1^		
Hindfoot Left	0.23	−0.15^1^	0.82	−0.06^1^	0.81	0.04^1^	0.38	−0.09^1^	0.67	−0.16^1^		
Hindfoot Right	0.95	0.01^1^	0.60	0.15^1^	0.63	0.09^1^	0.34	0.10^1^	0.09	−0.59^3^		
Left foot (%)	0.25	−0.15^1^	0.45	−0.21^2^	0.92	0.01^1^	0.20	−0.13^1^	0.73	0.13^1^		
Right foot (%)	0.37	0.12^1^	0.45	0.21^2^	0.93	0.01^1^	0.25	0.12^1^	0.73	−0.13^1^		
Forefoot (%)	0.21	0.17^1^	0.88	0.04^1^	0.85	−0.03^1^	0.73	0.03^1^	0.30	0.38^2^		
Hindfoot (%)	0.25	−0.15^1^	0.40	0.24^2^	0.85	0.03^1^	0.96	0.01^1^	0.35	−0.35^2^		
Frontal sway	0.35	0.12^1^	0.26	0.31^2^	0.39	0.17^1^	0.24	0.12^1^	**0.03**	0.71^4^		
Sagittal sway	0.62	0.06^1^	0.49	0.19^1^	0.77	0.05^1^	0.41	0.08^1^	0.18	0.48^3^		
Ellipse area	0.47	0.09^1^	0.14	0.40^3^	0.54	0.12^1^	0.27	0.11^1^	0.17	0.50^3^		
Ellipse width	0.41	0.10^1^	0.42	0.23^2^	0.86	0.03^1^	0.42	0.08^1^	0.06	0.66^4^		
Ellipse height	0.59	0.07^1^	**0.05**	0.53^3^	0.22	0.24^2^	0.14	0.15^1^	0.21	0.46^3^		

	Laterostrusion right						Laterotrusion left					
	Norm (50)		Hypofunctional (31)		Hyperfunctional (17)		Norm (60)		Hypofunctional (20)		Hyperfunctional (18)	
	p-value	Rho	p-value	Rho	p-value	Rho	p-value	Rho	p-value	Rho	p-value	Rho
Forefoot Left	0.44	0.11^1^	0.95	0.01^1^	0.22	−0.31^2^	0.75	0.04^1^	0.98	−0.003^1^	0.35	−0.23^2^
Forefoot Right	0.18	0.19^1^	0.80	−0.04^1^	0.77	−0.07^1^	**0.05**	0.25^2^	0.09	−0.38^2^	0.22	−0.29^2^
Hindfoot Left	0.68	−0.05^1^	0.41	−0.15^1^	0.79	0.06^1^	0.38	−0.11^1^	0.94	−0.01^1^	0.81	0.05^1^
Hindfoot Right	0.21	−0.17^1^	0.22	0.22^2^	0.63	0.12^1^	0.60	−0.06^1^	0.31	0.23^2^	0.23	0.29^2^
Left foot (%)	0.90	0.01^1^	0.32	−0.18^1^	0.43	−0.20^2^	0.23	−0.15^1^	0.52	0.14^1^	0.34	−0.23^2^
Right foot (%)	0.66	−0.06^1^	0.27	0.20^2^	0.43	0.20^2^	0.29	0.13^1^	0.52	−0.14^1^	0.34	0.23^2^
Forefoot (%)	0.16	0.20^2^	0.89	0.02^1^	0.57	−0.14^1^	0.10	0.21^2^	0.38	−0.20^2^	0.23	−0.29^2^
Hindfoot (%)	0.21	−0.17^1^	0.64	0.08^1^	0.57	0.14^1^	0.13	−0.19^1^	0.07	0.40^3^	0.22	0.30^2^
Frontal sway	**0.01**	0.34^2^	0.98	0.01^1^	0.24	−0.29^2^	0.08	0.22^2^	0.77	0.06^1^	0.42	−0.20^2^
Sagittal sway	**0.01**	0.33^2^	0.87	0.02^1^	0.22	−0.30^2^	0.08	0.22^2^	0.58	−0.13^1^	0.60	−0.13^1^
Ellipse area	**0.02**	0.32^2^	0.54	0.11^1^	0.25	−0.29^2^	0.12	0.20^2^	0.61	0.11^1^	0.29	−0.26^2^
Ellipse width	**0.01**	0.36^2^	0.48	0.13^1^	0.11	−0.39^2^	0.17	0.17^1^	0.97	−0.01^1^	0.42	−0.20^2^
Ellipse height	**0.05**	0.27^2^	0.35	0.17^1^	0.49	−0.17^1^	0.06	0.24^2^	0.22	0.28^2^	0.51	−0.16^1^

Calculations are omitted when the group size is < 10 subjects. Effect size classification (rho) according to Evans: 1 = < 0.2 (poor); 2 = 0.2–0.4 (weak); 3 = 0.4–0.6 (moderate); 4 = 0.6–0.8 (strong); 5 = > 0.8 (optimal). Significant correlations are marked in bold

In protrusion, participants with hypomobility showed a positive correlation between ellipse height and BMI (*p* ≤ 0.05; 95% CI −0.03- 1.00; moderate effect size), indicating that a higher BMI was associated with a greater ellipse height.

Similarly, in hypomobile mouth opening, BMI was positively correlated with frontal sway (*p* ≤ 0.03; 95% CI 0.13- 1.00; strong effect size), indicating increased sway with higher BMI.

In right laterotrusion, participants with normal mobility (*n* = 50) showed positive correlations between BMI and frontal sway (95% CI 0.07- −0.62), sagittal sway (95% CI 0.06- −0.61), ellipse area (95% CI 0.05–0.60), width (95% CI 0.10–0.64) and height (95% CI −0.01- 0.55), all with weak effect sizes (*p* ≤ 0.01, *p* ≤ 0.01, *p* ≤ 0.02, *p* ≤ 0.01 and *p* ≤ 0.05, respectively). All parameters increased with increasing BMI.

In left laterotrusion, right forefoot load increased with BMI in participants with normal mobility (*n* = 60) (*p* ≤ 0.05, 95% CI −0.01- 0.51, weak effect size).

Table 5 shows the mean, standard deviation, median, first and third quartiles, tolerance range (lower and upper limits) and confidence intervals (lower and upper bounds) for the parameters analysed by the model.

**Table 5:** Mean, standard deviation, median, first and third quartiles, tolerance range (lower and upper limits), and confidence interval (lower and upper bounds) of the model analysis parameters

	Mean	Standard deviation	Median	1. Quartille	3. Quartille	Tolerance range lower upper bounds bounds		Confidence interval Left Right bounds bounds	
Midline deviation maxilla (mm)	0.29	0.54	0.00	0.00	0.50	0.00	2.00	0.19	0.40
Midline deviation mandible (mm)	0.22	0.37	0.00	0.00	1.00	0.00	1.26	0.14	1.18
Supporting zone maxilla Right (mm)	1.59	2.40	0.60	0.17	1.55	0.00	8.21	1.10	2.07
Supporting zone maxilla Left (mm)	1.56	2.53	0.50	0.20	1.12	0.00	8.30	1.05	2.07
Supporting zone mandible Right (mm)	1.54	2.25	0.50	0.30	2.00	0.00	8.81	1.09	1.99
Supporting zone Left (mm)	1.51	2.02	0.90	0.40	1.57	0.00	8.25	1.10	1.91
Transverse width diff, maxilla (mm)	3.41	2.48	2.80	1.50	5.07	0.09	9.81	2.91	3.91
Transverse width diff, mandible (mm)	1.53	2.04	0.65	0.00	2.80	0.00	7.00	1.12	1.94
Okklusion first molar Right (mm)	2.37	2.72	1.25	0.00	4.25	0.00	8.00	1.83	2.92
Okklusion canine Right (mm)	1.71	2.16	0.40	0.00	3.50	0.00	7.00	1.27	2.14
Okklusion first molar Left (mm)	2.11	2.70	1.00	0.00	3.50	0.00	8.76	1.57	2.66
Okklusion canine Left (mm)	1.68	1.88	1.50	0.00	3.50	0.00	5.66	1.30	2.06
Overjet (mm)	3.93	1.83	4.00	3.00	5.00	0.00	8.00	3.57	4.30
Overbite (mm)	3.44	1.79	3.50	2.00	5.00	0.00	7.00	3.08	3.80

Correlations between canine (3s) and first molar (6s) occlusion on the left and right sides, and postural control parameters, showed the following results:

For the right canines, a significant positive correlation was found with frontal sway (*p* ≤ 0.05; 95% CI −0.01–0.39; effect size: poor), as well as with ellipse area (*p* ≤ 0.05; 95% CI 0.01–0.39; effect size: poor) and width (*p* ≤ 0.01; 95% CI 0.05–0.44 effect size: poor).

For the left canines, a positive significant correlation was observed with sagittal sway (*p* ≤ 0.01; 95% CI 0.05–0.44; effect size poor) and with ellipse area (*p* ≤ 0.02; 95% CI 0.02–0.42; effect size poor) and width (*p* ≤ 0.01; 95% CI 0.07–0.46; effect size poor).

For the right first molars, positive significant correlations were identified with frontal and sagittal sway (*p* ≤ 0.01 (95% CI 0.09–0.47) and 0.03 (95% CI 0.02–0.41), respectively; effect size: poor), as well as with ellipse area and width (*p* ≤ 0.01 (95% CI 0.07–0.46) and 0.001 (95% CI 0.12–0.50), respectively; effect size: poor to weak).

A significant association was found between overbite and the right foot (*p* ≤ 0.04; 95% CI 0.00- 0.40; effect size: poor).

No significant correlations were found for the left first molars, transverse width discrepancies in the maxilla and mandible, midline deviations in the maxilla and mandible, or overjet with any postural control parameters Table 6.

**Table 6:** Summary of significant correlations between model analysis parameters and postural parameters. Calculations are omitted when the group size is < 10 subjects

	Okklusion canine right		Okklusion canine left		Okklusion first molar right		Overbite (vertikal)		Overjet (horizontal)	
	*p*-value	rho	*p*-value	rho	*p*-value	rho	*p*-value	rho	*p*-value	rho
Forefoot left (%)	0.93	0.01	0.92	0.01	0.27	0.11	0.19	0.13	0.84	0.02
Forefoot right (%)	0.27	0.11	0.54	0.06	0.40	0.05	0.76	0.03	0.27	0.11
Hindfoot left (%)	0.54	0.06	0.18	0.13	0.67	0.04	0.34	0.09	0.64	0.04
Hindfoot right (%)	0.15	0.14	0.45	0.07	0.82	0.02	0.17	0.13	0.57	0.05
Left (%)	0.85	0.01	0.12	0.15	0.58	0.05	0.06	0.19	0.50	0.06
Right (%)	0.93	0.01	0.10	0.16	0.71	0.03	**0.04**	0.20	0.40	0.08
Forefoot (%)	0.28	0.10	0.65	0.04	0.93	0.007	0.60	0.05	0.39	0.08
Hindfoot (%)	0.40	0.08	0.77	0.02	0.80	0.02	0.39	0.08	0.68	0.04
Frontal sway (%)	**0.05**	0.19	0.14	0.14	**0.01**	0.28	0.99	0.01	0.60	0.05
Sagittal sway (%)	0.06	0.18	**0.01**	0.24	**0.03**	0.21	0.61	0.05	0.23	0.12
Ellipse area	**0.05**	0.19	**0.02**	0.22	**0.01**	0.26	0.82	0.02	0.44	0.07
Ellipse width	**0.01**	0.24	**0.01**	0.26	**0.001**	0.31	0.68	0.04	0.33	0.09
Ellipse height	0.16	0.14	0.10	0.16	0.06	0.18	0.77	0.02	0.35	0.09

Effect size classification (rho) according to Evans: 1 = < 0.2 (poor); 2 = 0.2–0.4 (weak); 3 = 0.4–0.6 (moderate); 4 = 0.6–0.8 (strong); 5 = > 0.8 (optimal). Significant correlations are marked in bold

Table 7 shows the significant group comparisons for the difference in transverse width of the maxilla and the occlusion of the right first molars, in relation to parameters of postural control.

**Table 7:** Summary of significant group comparisons of the transverse width difference in the maxilla and the occlusion of the right first molars with respect to postural parameters

	Transversal width difference maxilla			Occlusion first molar right		
	*p*-value	Group comparisom	*p*-Bonf-Holm	*p*-value	Group comparisom	*p*-Bonf-Holm
Forefoot Left	0.15			0.17		
Forefoot Right	0.77			0.75		
Hindfoot Left	**0.02**	**1 vs. 2** **1 vs. 3**	**0.05** **0.02**	0.82		
Hindfoot Right	0.45			0.34		
Left foot (%)	0.74			0.60		
Right foot (%)	0.92			0.59		
Forefoot (%)	0.09			0.26		
Hindfoot (%)	0.27			0.29		
Frontal sway	**0.001**	**1 vs. 2** **2 vs. 3**	**0.01** **0.04**	**0.01**	**1 vs. 3**	**0.01**
Sagittal sway	**0.01**	**1 vs. 2** **2 vs. 3**	**0.04** **0.001**	**0.02**	**1 vs. 3**	**0.02**
Ellipse area	**0.001**	**1 vs. 2** **2 vs. 3**	**0.02** **0.02**	**0.01**	**1 vs. 3**	**0.01**
Ellipse width	**0.001**	**1 vs. 2** **2 vs. 3**	**0.01** **0.01**	**0.001**	**1 vs. 3**	**0.01**
Ellipse height	**0.01**	**1 vs. 2** **2 vs. 3**	**0.05** **0.05**	**0.04**	**1 vs. 3**	**0.04**

Calculations are omitted when the group size is < 10 subjects. Significant correlations are marked in bold

For the transverse width difference in the maxilla, the group comparison shows an overall p-value of *p* ≤ 0.02 for the left hindfoot, with differences between groups 1 and 2 (*p* ≤ 0.05) and groups 1 and 3 (*p* ≤ 0.02). For the parameters of frontal and sagittal sway, ellipse area, height and width, the overall p-values were found to be *p* ≤ 0.001 and *p* ≤ 0.01, followed by group differences between groups 1 and 2, and groups 2 and 3 (*p* ≤ 0.001–0.05).

For the occlusion of the right first molars, the overall p-values for the aforementioned parameters range from *p* ≤ 0.001 to 0.04, with subsequent group differences between groups 1 and 3 (*p* ≤ 0.01–0.04).

In the support zone of the left maxilla, a significant overall p-value was found (*p* ≤ 0.03), but no significant Conover–Iman post hoc comparisons were detected. A similar pattern was observed in the left hindfoot (*p* ≤ 0.04) and in sagittal sway and ellipse width (*p* ≤ 0.02) in the occlusion of the left first molars, as no significant Conover–Iman comparisons were detected.

No significant overall p-values were found for midline deviations in the maxilla and mandible, the support zones of the right maxilla and mandible, the support zone of the left mandible, the occlusion of the right canines or left first molars, or for overbite and overjet.

Table 8 presents the correlations between the occlusal parameters obtained from the model analysis and the postural parameters, which are categorised by BMI groups (normal weight, pre-obese and obese). There are some isolated significant findings:

**Table 8:** Correlation between the occlusal parameters from model analysis and postural parameters, filtered by BMI groups (normal weight, pre-obese, obese)

	Occlusion first molar right						Occlusion first molar left					
	Normal weight (36)		Pre-obese (45)		Obese (17)		Normal weight		Pre-obese		Obese	
	p-value	Rho	p-value	Rho	p-value	Rho	p-value	Rho	p-value	Rho	p-value	Rho
Forefoot Left	0.08	0.29	0.15	0.21	0.44	−0.19	0.76	−0.05	0.54	−0.09	0.78	0.07
Forefoot Right	**0.04**	0.34	**0.01**	−0.39	0.85	−0.04	0.80	−0.04	0.18	0.20	0.68	0.10
Hindfoot Left	0.43	−0.13	0.18	0.19	0.62	0.12	0.19	0.21	0.15	−0.21	0.39	−0.21
Hindfoot Right	0.09	−0.28	0.62	0.07	0.15	0.36	0.88	0.02	0.54	0.09	0.16	0.35
Left foot (%)	0.40	0.14	0.13	0.22	0.25	−0.28	0.64	0.07^2^	0.08	−0.26^2^	0.31	−0.25
Right foot (%)	0.51	−0.11	0.21	−0.18	0.25	0.28	0.59	−0.09^2^	0.06	0.28^2^	0.31	0.25
Forefoot (%)	0.06	0.31	0.11	−0.23	0.79	−0.06	0.52	−0.11^2^	0.35	0.13^2^	0.64	0.11
Hindfoot (%)	0.12	−0.25	0.11	0.23	0.96	−0.01	0.34	0.16^2^	0.37	−0.13^2^	0.37	−0.22
Frontal sway	**0.02**	0.34	0.40	0.12	0.07	0.42	0.61	0.08^2^	0.62	−0.07^2^	0.81	0.06^2^
Sagittal sway	**0.04**	0.31	0.88	0.02	0.07	0.43	0.92	0.01^2^	0.88	−0.02^2^	0.85	0.04
Ellipse area	**0.01**	0.38	0.89	0.02	**0.04**	0.47	0.22	0.21^2^	0.89	−0.01^2^	0.67	0.10
Ellipse width	**0.01**	0.42	0.36	0.13	0.07	0.43	0.16	0.23	0.98	−0.002	0.57	0.14
Ellipse height	**0.03**	0.32	0.51	−0.09	**0.001**	0.65	0.77	0.04	0.88	−0.02	0.35	0.24

	Occlusion canine reight						Occlusion canine left					
	Normal weight		Pre-obese		Obese		Normal weight		Pre-obese		Obese	
	p-value	Rho	p-value	Rho	p-value	Rho	p-value	Rho	p-value	Rho	p-value	Rho
Forefoot Left	0.24	−0.19	0.20	0.19	0.62	−0.12	0.82	0.03	0.54	−0.09	0.83	0.05
Forefoot Right	0.19	0.22	0.13	−0.22	0.20	−0.32	0.45	0.12	0.89	0.01	0.90	0.02
Hindfoot Left	0.09	0.28	0.70	−0.05	0.95	−0.01	0.54	0.10	**0.03**	−0.31	0.86	−0.04
Hindfoot Right	0.76	−0.05	0.34	0.14	**0.01**	0.62	0.48	−0.12	0.21	0.18	0.58	0.14
Left foot (%)	0.95	0.01	0.81	0.03	0.38	−0.21	0.82	0.03	0.07	−0.26	0.84	−0.05
Right foot (%)	0.98	−0.01	0.65	−0.06	0.38	0.21	0.71	−0.06	**0.02**	0.31	0.84	0.05
Forefoot (%)	0.42	−0.13	0.74	−0.04	0.36	−0.22	0.55	0.10	0.86	0.02	0.20	0.04
Hindfoot (%)	0.52	0.11	0.80	0.03	0.46	0.18	0.84	−0.03	0.84	−0.0^2^	0.61	−0.12
Frontal sway	**0.01**	0.39	0.50	0.10	0.93	0.02	0.31	0.17	0.25	0.17	0.95	−0.01
Sagittal sway	**0.03**	0.34	0.37	0.13	0.91	0.02	0.13	0.25	0.26	0.17	0.59	0.13
Ellipse area	**0.03**	0.33	0.32	0.14	0.96	−0.01	**0.10**	0.27	0.06	0.28	0.87	0.03
Ellipse width	**0.01**	0.39	0.17	0.20	0.79	0.06	**0.03**	0.32	0.08	0.26	0.41	0.20
Ellipse height	0.10	0.27	0.55	0.09	0.67	0.10	0.27	0.18	0.18	0.19	0.72	0.09

	Transverse width diff, maxilla						Transverse width diff, mandible					
	Normal weight (36)		Pre-obese (45)		Obese (17)		Normal weight (36)		Pre-obese (45)		Obese (17)	
	p-value	Rho	p-value	Rho	p-value	Rho	p-value	Rho	p-value	Rho	p-value	Rho
Forefoot Left	0.48	0.11	0.73	0.05	0.06	0.47	0.70	0.06	0.20	−0.19	**0.01**	0.59
Forefoot Right	0.59	0.09	0.49	−0.10	0.56	−0.14	0.75	0.05	0.84	0.02	0.86	0.04
Hindfoot Left	0.95	0.01	0.70	0.05	0.30	−0.26	0.64	−0.08	0.65	−0.06	0.25	−0.29
Hindfoot Right	0.29	−0.17	0.85	−0.02	0.33	−0.25	0.80	−0.04	0.45	0.11	**0.01**	−0.62
Left foot (%)	0.71	0.06	0.85	−0.02	0.25	0.29	0.79	0.04	0.15	−0.21	**0.01**	0.56
Right foot (%)	0.49	−0.11	0.87	0.02	0.25	−0.29	0.82	−0.03	0.22	0.18	**0.02**	−0.56
Forefoot (%)	0.31	0.17	0.76	−0.04	0.29	0.26	0.36	0.15	0.82	−0.03	0.06	0.47
Hindfoot (%)	0.61	−0.08	0.77	0.04	0.54	−0.15	0.37	−0.15	0.84	0.02	0.20	−0.32
Frontal sway	0.92	0.01	0.60	−0.07	0.58	−0.14	0.33	0.16	0.66	−0.06	0.81	−0.05
Sagittal sway	0.76	−0.05	0.17	−0.20	0.98	−0.01	0.41	0.14	0.42	0.12	0.84	−0.05
Ellipse area	0.92	0.01	0.10	−0.24	0.59	−0.13	0.18	0.22	0.78	−0.04	0.58	−0.14
Ellipse width	0.78	−0.04	0.18	−0.20	0.92	0.02	0.47	0.12	0.97	0.005	0.76	−0.07
Ellipse height	0.67	0.07	0.09	−0.25	0.16	−0.35	0.14	0.24	0.84	−0.03	0.17	−0.34

	Midline deviation maxilla						Midline deviation mandible					
	Normal weight (36)		Pre-obese (45)		Obese (17)		Normal weight (36)		Pre-obese (45)		Obese (17)	
	p-value	Rho	p-value	Rho	p-value	Rho	p-value	Rho	p-value	Rho	p-value	Rho
Forefoot Left	0.92	0.01	0.49	0.10	0.80	−0.06	0.76	−0.05	0.35	−0.13	0.81	0.06
Forefoot Right	0.33	0.16	0.80	0.03	0.06	0.46	0.73	0.05	0.41	0.12	0.78	−0.07
Hindfoot Left	0.99	−0.01	0.31	−0.15	0.56	−0.14	0.55	−0.10	0.47	−0.10	0.78	0.07
Hindfoot Right	0.74	0.05	0.52	0.09	0.61	−0.13	0.54	0.10	0.79	0.03	0.89	0.03
Left foot (%)	0.84	0.03	0.55	−0.09	0.55	−0.15	0.24	−0.19	0.50	−0.10	0.81	−0.06
Right foot (%)	0.76	−0.05	0.33	0.14	0.55	0.15	0.31	0.17	0.55	0.09	0.81	0.06
Forefoot (%)	0.77	0.04	0.45	0.11	0.28	0.27	0.90	0.01	0.80	−0.03	0.50	0.17
Hindfoot (%)	−0.91	0.01	0.48	−0.10	0.42	−0.20	0.88	0.02	0.80	0.03	0.85	−0.04
Frontal sway	0.53	−0.10	**0.03**	0.30	0.25	0.28	0.61	0.08	0.47	−0.10	0.54	0.15
Sagittal sway	0.49	−0.11	0.71	0.05	0.07	0.44	0.39	0.14	0.77	0.04	0.96	−0.01
Ellipse area	0.46	−0.12	0.07	0.26	0.24	0.29	0.48	0.11	0.75	−0.04	0.63	0.12
Ellipse width	0.75	−0.05	0.44	0.11	0.06	0.45	0.99	−0.002	0.56	−0.08	0.78	−0.07
Ellipse height	0.41	−0.13	0.08	0.26	0.67	0.11	0.31	0.17	0.87	−0.02	0.29	0.26

Effect size classification (rho) according to Evans: 1 = < 0.2 (poor); 2 = 0.2–0.4 (weak); 3 = 0.4–0.6 (moderate); 4 = 0.6–0.8 (strong); 5 = > 0.8 (optimal). Significant correlations are marked in bold

For occlusion of the right first molars, the normal weight group showed positive correlations with the right forefoot (*p* ≤ 0.04; 95% CI −0.01- 0.66), frontal (*p* ≤ 0.02; 95% CI 0.05–0.71) and sagittal sway (*p* ≤ 0.04; 95% CI 0.02–0.68) and ellipse area (*p* ≤ 0.01; 95% CI 0.10–0.74), width (*p* ≤ 0.01; 95% CI 0.15–0.78) and height (*p* ≤ 0.03; 95% CI 0.04–0.69), all with weak effect sizes. In the pre-obese group, only a significant negative correlation with the right forefoot (*p* ≤ 0.01; 95% CI −0.68- −0.11) with a weak effect size was observed.

For the occlusion of the right canines, the normal-weight group shows weak significant correlations with frontal and sagittal sway (*p* ≤ 0.01 (95% CI 0.07–0.72) and *p* ≤ 0.03 (95% CI 0.06–0.71), respectively) and with ellipse area (*p* ≤ 0.03; 95% CI 0.04–0.70) and width (*p* ≤ 0.01; 95% CI 0.12–0.76). In the obese group, a significant positive correlation was observed with the right hindfoot (*p* ≤ 0.01; 95% CI 0.22- 1.00) with a strong effect size.

Regarding the occlusion of the left canines, significant correlations were found in the normal-weight group with ellipse width (*p* ≤ 0.03; 95% CI 0.07–0.72) and in the pre-obese group with the left hindfoot (*p* ≤ 0.0395% CI −0.61- −0.03) and right foot (*p* ≤ 0.03; 95% CI 0.03–0.61) all with weak effect sizes.

For the transverse width difference in the mandible, significant correlations were observed in the obese group with the left forefoot (95% CI 0.18- 1.00), right hindfoot (95% CI −1.00 − 0.21) and both feet (left: *p* ≤ 0.01; 95% CI 0.14- 1.00 and right: *p* ≤ 0.02; 95% CI −1.00−0.14), all of which had moderate to strong effect sizes.

Regarding midline deviation in the maxilla, only one positive correlation with frontal sway (*p* ≤ 0.03; 95% CI 0.01–0.60) with a weak effect size was found in the pre-obese group.

No significant correlations were found in any BMI group for the occlusion of the left first molars, the difference in transverse width in the maxilla or the midline deviation in the mandible.

Table 9 summarises the correlations between BMI and postural control, stratified by model analysis group.

**Table 9:** Summary of significant correlations between BMI and postural parameters, filtered by model analysis groups (neutral, distal, mesial) of the measured parameter

	Occlusion first molar right		Occlusion canine right				Occlusion first molar left			
	mesial (16)		neutral (49)		distal (45)		neutral (49)		distal (45)	
	p-value	Rho	p-value	Rho	p-value	Rho	p-value	Rho	p-value	Rho
Forefoot Left	**0.001**	−0.69	0.15	−0.20	0.18	0.20	0.74	0.04	0.93	−0.01
Forefoot Right	0.98	−0.01	0.19	0.18	0.39	−0.12	0.95	0.01	0.17	0.21
Hindfoot Left	**0.01**	0.65	0.13	0.21	**0.03**	−0.31	0.46	0.10	**0.01**	−0.36
Hindfoot Right	0.35	0.24	0.52	−0.09	0.18	0.20	0.60	−0.07	0.23	0.18
Left foot (%)	0.31	−0.26	0.66	−0.06	0.22	−0.18	0.57	0.08	0.06	−0.29
Right foot (%)	0.31	0.26	0.63	0.06	0.35	0.14	0.37	−0.13	0.06	0.29
Forefoot (%)	**0.03**	−0.55	0.79	−0.03	0.29	0.15	0.63	0.07	0.34	0.14
Hindfoot (%)	**0.03**	0.55	0.63	0.06	0.55	−0.08	0.80	0.03	0.33	−0.15
Frontal sway	0.74	0.08	**0.03**	0.30	0.50	0.10	**0.04**	0.30	0.64	0.07
Sagittal sway	0.72	−0.09	0.13	0.21	0.34	0.14	0.21	0.18	0.66	0.06
Ellipse area	0.73	0.09	0.07	0.25	0.45	0.11	0.08	0.25	0.59	0.08
Ellipse width	0.62	−0.13	0.16	0.20	0.40	0.12	0.08	0.25	0.83	0.03
Ellipse height	0.53	0.16	**0.03**	0.30	0.34	0.14	0.08	0.25	0.39	0.13

Calculations are omitted when the group size is < 10 subjects. Effect size classification (rho) according to Evans: 1 = < 0.2 (poor); 2 = 0.2–0.4 (weak); 3 = 0.4–0.6 (moderate); 4 = 0.6–0.8 (strong); 5 = > 0.8 (optimal). Significant correlations are marked in bold

In the mesial group of the right first molar, BMI shows significant negative and positive correlations with the left forefoot (*p* ≤ 0.001; 95% CI −1.00 - −0.29) and left hindfoot (*p* ≤ 0.01; 95% CI 0.22- 1.00), respectively, with strong effect sizes. Moderate significant positive and negative correlations (*p* ≤ 0.03) were found for the combined forefoot (95% CI −1.00 - −0.08) and hindfoot (95% CI 0.08–1.00).

Regarding the occlusion of the left first molars, the neutral group exhibits a significant positive correlation between BMI and frontal sway (*p* ≤ 0.04; 95% CI 0.01–0.58; weak effect size), whereas the distal group exhibits a significant negative correlation with the left hindfoot (*p* ≤ 0.01; 95% CI 0.66- −0.06; weak effect size).

For occlusion of the right canines, the neutral group showed significant positive correlations between BMI and frontal sway (*p* ≤ 0.03; 95% CI 0.02–0.58; weak effect size), as well as ellipse height (*p* ≤ 0.03; 95% CI 0.04–0.60; weak effect size). In the distal group, a significant negative correlation was observed between BMI and the left hindfoot (*p* ≤ 0.03; 95% CI −0.61 - −0.03; weak effect size).

## Discussion

The present study aimed to analyse the interdependencies between dental parameters, mandibular mobility and static postural sway as well as load distribution in male subjects of advanced working age. The 98 participants, who perceived themselves as healthy, were predominantly overweight to mildly obese according to their BMI (mean BMI: 27 ± 4 kg/m²) and exhibited balanced weight distribution between the left (51%) and right (49%) sides of their body. The forefoot–hindfoot ratio (38:62) corresponded to values reported in other studies involving normal-weight and pre-obese adults [25, 35].

Similarly, median postural sway of 14 mm in the medio-lateral direction and 20 mm in the antero-posterior direction, with an ellipse area of 22.24 cm², falls within the normal range for healthy middle-aged to older adults of normal weight or with moderate obesity (BMI 25–35 kg/m²) described in the literature [25, 35, 69–72]. Axiographically measured laterotrusion to the left and right, mouth opening and protrusion were within normal ranges for the majority of participants. Mandibular mobility parameters (laterotrusion, protrusion, mouth opening) were within normal ranges for the majority of participants [22, 23]. In contrast, dental model analysis revealed that none of the participants exhibited Angle Class I occlusion: 75% were classified as Class II and 25% as Class III. Heren, the mean overjet (3.93 mm) and mean overbite (3.44 mm) both exceeded slightly the reference ranges of 2.1–2.9 mm and 1–3 mm, respectively, as defined by the Schopf classification [65, 66] This indicates that while mandibular function was largely preserved, dental alignment in this cohort of men aged 51 to 60 deviated substantially from ideal norms, consistent with the low proportion who had undergone orthodontic treatment (21%). These findings indicate that the cohort was characterized by prevalent dental malalignment despite largely normal mandibular function [22, 23]. 

Furthermore, postural control parameters were not influenced by physical activity, orthodontic treatment or BMI across all participants. With small effect sizes, increasing body height was associated with increased hindfoot loading, greater sagittal sway and greater ellipse width.

No significant correlations were found between axiographic (mouth opening, protrusion and laterotrusion to the left and right) and postural sway and load distribution.

A more detailed subgroup analysis based on normative classifications of mandibular mobility revealed that leftward laterotrusion and mouth opening did not affect postural stability. However, the opposite was true for laterotrusion to the right and protrusion: participants with laterotrusion to the right within the normative range (7–12 mm) exhibited a shift in load from the right foot to the left foot, as well as an increase in ellipse area, with increasing laterotrusion. For protrusion, changes were only observed in men with hyperfunctional (above normal) or hypofunctional (below normal) mandibular movement. In hypofunctional protrusion, an increase in hyperfunction was associated with an increase in ellipse height, whereas an increase in hypofunction led to greater sagittal sway.

The most significant correlations between the model analysis and parameters of postural sway and load distribution were observed in occlusion, particularly with regard to the relationships between the canines and the first molars. However, classification according to Angle classes (Classes II [II:1 + II:2] and III) had no significant impact on postural parameters (*p* > 0.05). Deviations in canine occlusion from ideal interdigitation were associated with increased frontal and sagittal sway and a larger sway ellipse on both the right and left sides. For example, these relationships were evident in the occlusion of the right first molar, where marked deviations from the physiological norm (class I relationship) (e.g. hyperocclusion > 2 mm or hypoocclusion < − 2 mm) were associated with increased frontal and sagittal sway, as well as a larger ellipse area and width. Furthermore, an increased overbite was associated with an increased load on the right foot.

However, it should be noted that most correlations showed weak to poor effect sizes and should therefore be interpreted cautiously as trends with limited clinical relevance.

The present results differ from findings in women aged 21–30 and 41–50 years using a similar study design [33]. In contrast, older men showed more complex patterns. Here, BMI did not show a general association with postural sway and load distribution, however some BMI stratified subgroup analyses showed associations with postural components in combination with certain mandibular or occlusal variables.

Systematic reviews [22–24] support the idea that malocclusions can negatively impact postural stability, demonstrating weak to moderate effects and showing that different occlusal positions can influence postural control, particularly centre-of-pressure displacement and ellipse parameters. Changes in mandibular position are reported to primarily affect medio-lateral sway, while malocclusions have a stronger influence on antero-posterior sway. However, the present findings do not confirm these conclusions, regardless of whether mandibular movement patterns or static occlusion are emphasised. Instead, effects were observed in both antero-posterior and medio-lateral sway (along with ellipse changes), while stance pattern (percentage load distribution) remained largely unchanged.

BMI has not previously been considered as a potential influencing factor in systematic reviews on this topic [22–25]. However, Hirjaková et al. [35] and Fischer et al. [37] reported negative effects of BMI on postural control in mildly obese adults, and Teasdale et al. [41] demonstrated that weight loss improves postural stability linearly. Ku et al. [36] also found increased mediolateral instability in men with abdominal obesity.

Based on these findings, the present data were analysed further in BMI-stratified subgroups exploratory. Neither correlation analyses nor group comparisons across BMI categories (normal weight, pre-obese and obese) revealed significant differences in postural control parameters such as postural sway and load distribution.

Within the normal-weight subgroup (*n* = 36), axiographic analysis showed that greater mouth opening was associated with significantly higher loading of the right foot. Model analysis indicated that malocclusion of the right first molar (with a deviation of less than 2 mm) combined with canine malocclusion was significantly associated with increased ellipse parameters (area, width and height) and increased frontal and sagittal sway (with weak effect sizes). Only the ellipse area and width increased on the left side with greater horizontal canine distance (weak effect sizes). Only model analysis showed correlations in pre-obese participants: an increase in the distance of the left canine was associated with a reduction in left hindfoot loading and an increase in right foot loading and sagittal sway (weak effect sizes). Additionally, frontal sway increased with greater midline deviation (weak effect size). In the 17 obese men (BMI ≥ 30 kg/m²), sagittal sway, ellipse area and width decreased with an increased range of right laterotrusion. Most correlations had weak effect sizes and should be interpreted as trends rather than clinically relevant findings. Consistent with the previous findings, the small effect sizes observed in this analysis should be considered when interpreting the results, as they suggest limited clinical relevance. Regarding occlusion, greater molar deviation from the ideal position was associated with increased ellipse area and height (moderate to strong effect sizes), while greater canine deviation was associated with increased right hindfoot loading (strong effect size). Additionally, greater differences in transverse mandibular width were associated with a shift in load towards the left forefoot, the right hindfoot and both feet overall (moderate to strong effect sizes). These findings suggest that local malocclusions, particularly in the right canine region and transverse mandibular discrepancies, are associated with measurable changes in load distribution and postural stability, particularly in obese men.

This asymmetric distribution of findings may reflect a genuine functional lateralization; however, it may also be influenced by the exploratory nature of the analyses and the number of statistical comparisons performed, which increases the possibility of chance findings. Given that 86% of participants in our cohort were right-handed, a potential contribution of functional dominance and habitual asymmetrical loading cannot be excluded. Previous studies [73–76] have suggested associations between handedness, masticatory preference, and asymmetrical neuromuscular function, which could provide a possible explanation for the observed right-sided predominance. Nevertheless, as chewing preference, masticatory muscle activity, and lateralisation of postural strategies were not assessed in the present study, this interpretation remains hypothetical.

The relationship between BMI and axiographic or model parameters was not linear across the full sample. Some associations became visible only in subgroup analyses, which suggests a possible interaction that should be examined in further investigations. The more detailed subgroup analysis, which combined BMI categories with axiographic classifications of mandibular mobility (normal, hypofunctional or hyperfunctional), showed that, in men with right laterotrusion within the physiological range (7–12 mm), a higher BMI was significantly associated with increased frontal and sagittal body sway, as well as larger sway ellipse parameters. In groups with functional impairments, a higher BMI combined with greater lateral deviation resulted in an increased sagittal displacement of the sagittal sway, as well as a larger ellipse area and height. Again, although statistically significant, these effects were predominantly small and should be interpreted as trends.

Overall, the findings suggest that future studies should investigate BMI, occlusal characteristics, and mandibular mobility within an integrated framework rather than as isolated factors. While BMI was not generally associated with postural control parameters in this cohort, the observed subgroup differences should be interpreted as preliminary and hypothesis-generating findings rather than evidence of a modifying effect. In summary, BMI did not show a general association with postural control parameters in this cohort, and the subgroup findings should be regarded as preliminary observations rather than evidence of a modifying effect.

Here, as limiting factor the cross-sectional design has to be considered as it only allows correlations to be identified, not causal relationships. Some variables, such as perceived health status and physical activity, were self-reported and may therefore be subject to bias. Furthermore, the inclusion of only asymptomatic men who subjectively perceive themselves to be healthy limits the generalizability of these findings to clinical populations with manifest temporomandibular disorders or chronic pain. A further methodological limitation concerns the assessment of participants’ health status and dental characteristics. Although removable dental prostheses were defined as exclusion criteria, other relevant dental factors, including fixed prosthodontic restorations (e.g., crowns, bridges, and implants), missing teeth, and extensive restorative treatments, were not systematically recorded. Moreover, health status was based on self-reported information and therefore reflects participants’ subjective perception of being healthy rather than an objective clinical evaluation. Consequently, undiagnosed or subclinical conditions and unrecorded dental alterations cannot be completely excluded.

Regarding the distribution of Angle classifications, it should be considered that the cohort was not intended to represent the prevalence of different occlusal classes in the general population, but rather to investigate potential associations between occlusal characteristics, mandibular function, and postural control. The absence of participants with Angle Class I occlusion and the predominance of Class II relationships may reflect characteristics of patients attending general dental practices rather than asystematic selection bias. Nevertheless, this limited representation of occlusal categories may restrict the generalizability of the findings to populations with a broader range of occlusal relationships. Previous restorative dental treatments, which are relatively common in individuals aged 51–60 years, may have influenced occlusal contact patterns, especially at the canine and first molar sites investigated in this study. As these factors were not systematically documented, their potential contribution to the observed associations between occlusal parameters and postural control remains uncertain. Future studies should include comprehensive dental examinations, including assessment of restorations, missing teeth, implant-supported prostheses, and objective health parameters, to better account for potential confounding factors affecting occlusal and postural outcomes.

A limitation of the present study is that some stratified subgroup analyses (e.g., according to BMI categories) were based on relatively small sample sizes, which may have reduced statistical power and increased the uncertainty of the estimated associations. Therefore, findings from these subgroup analyses, particularly strong correlations observed in small subgroups, should be interpreted with caution.

Future longitudinal or interventional studies should examine whether the observed associations are reproducible and whether they have any practical relevance. It should also be noted that the present study design used only a single static condition (eyes open, habitual jaw position). The standard paradigm for probing functional links between the occlusal system and postural control typically includes additional conditions, such as clenching versus rest, or eyes-closed trials, which increase postural demand and make any occlusion-related effects more detectable [22, 23]. By contrast, correlating static dental morphology with postural control under a single, low-demand condition represents a conservative and indirect test of a functional relationship. The absence of an eyes-closed condition means that visual compensation for any postural relevant input from the temporomandibular system could not be assessed. Future studies should incorporate task manipulation (e.g., clenching, protrusion) and visual condition variations to provide a more direct test of the mechanisms hypothesized here.

The findings of this study are primarily applicable to healthy, asymptomatic men aged 51–60 years. Therefore, the generalizability of the results to women, other age groups, or clinical populations with temporomandibular disorders or other musculoskeletal conditions is limited. Future studies including more diverse populations are warranted to determine whether the observed associations are consistent across different demographic and clinical groups.

## Conclusion

Therefore, clinical relevant association between postural sway and load distribution and occlusion, or mandibular mobility parameter when analyzed across the full sample of 98 men aged 51 to 60 years. In addition, static postural control parameters were not influenced by physical activity, orthodontic treatment or BMI across all participants. Additional exploratory subgroup analyses indicated that deviations in canine and molar occlusion were associated with increased postural sway and larger sway ellipses. An increased BMI does not appear to directly cause postural instability but may be associated with, or contribute to, pre-existing functional limitations or occlusal dysfunctions. Therefore, clinical assessment should adopt a holistic approach that considers BMI alongside individual constitutional, occlusal, and functional parameters.

The cross-sectional design limits interpretation to associations rather than causal relationships. Additionally, the inclusion of only healthy, asymptomatic men limits generalizability to clinical populations, while unrecorded factors such as restorative treatments, missing teeth, implant-supported prostheses, and the use of a single static posturographic condition (eyes open, habitual jaw position) may have influenced the observed relationships.

## Supplementary Information

Below is the link to the electronic supplementary material.

Supplementary Material 1 (PDF 192 KB)

## Data Availability

Data is provided within the manuscript.
